# Factors Affecting the Pharmacology of Antibody–Drug Conjugates

**DOI:** 10.3390/antib7010010

**Published:** 2018-02-07

**Authors:** Andrew T. Lucas, Lauren S. L. Price, Allison N. Schorzman, Mallory Storrie, Joseph A. Piscitelli, Juan Razo, William C. Zamboni

**Affiliations:** 1 Division of Pharmacotherapy and Experimental Therapeutics, UNC Eshelman School of Pharmacy, University of North Carolina at Chapel Hill, Chapel Hill, NC 27599, USA; andrew_lucas@unc.edu (A.T.L.); lslprice@email.unc.edu (L.S.L.P.); aschorz@email.unc.edu (A.N.S.); 2 UNC Eshelman School of Pharmacy, Chapel Hill, NC 27599, USA; mastorrie@gmail.com (M.S.); joseph_piscitelli@unc.edu (J.A.P.); juanrazo@email.unc.edu (J.R.); 3 Lineberger Comprehensive Cancer Center, University of North Carolina at Chapel Hill, Chapel Hill, NC 27599, USA

**Keywords:** antibody–drug conjugates, mononuclear phagocyte system, pharmacokinetics, pharmacology, therapeutic proteins

## Abstract

Major advances in therapeutic proteins, including antibody–drug conjugates (ADCs), have created revolutionary drug delivery systems in cancer over the past decade. While these immunoconjugate agents provide several advantages compared to their small-molecule counterparts, their clinical use is still in its infancy. The considerations in their development and clinical use are complex, and consist of multiple components and variables that can affect the pharmacologic characteristics. It is critical to understand the mechanisms employed by ADCs in navigating biological barriers and how these factors affect their biodistribution, delivery to tumors, efficacy, and toxicity. Thus, future studies are warranted to better understand the complex pharmacology and interaction between ADC carriers and biological systems, such as the mononuclear phagocyte system (MPS) and tumor microenvironment. This review provides an overview of factors that affect the pharmacologic profiles of ADC therapies that are currently in clinical use and development.

## 1. Introduction

The number of available carrier-based drug systems for the treatment of cancer and other diseases has seen exponential growth in the past three decades. As of 2013, there are more than 1600 nanotechnology-based products in the market, and almost 250 nanomedicine agents on the market or in clinical trials, with more emerging at a rapid pace [1]. In addition, within the past 20 years, the use and approval of monoclonal antibodies (mAbs) has risen sharply, both in the clinic as well as in development, to advance a revolution in immunotherapy. While early mAb therapies were plagued with toxicities due to immunogenicity, modern genetic engineering has led to the human/humanized antibodies that we use today [2]. Research into antibody–drug conjugates (ADCs), conjugating highly potent cytotoxic agents to targeted mAbs, has become a very active area of drug development for the treatment of cancer. There are currently ~50 ADCs in 125 clinical trials with ~35 different ADC formulations in >50 phase I/II studies in patients with solid tumors (Table 1) [3].

Even though these ADCs have been used for nearly two decades, there is still much to learn about the factors that affect the disposition of ADCs and antibody-based therapies. Understanding the factors affecting pharmacokinetic (PK) and pharmacodynamic (PD) variability, the exposure–response relationship, and evaluating potential methods to individualize therapy of ADCs are essential to increasing their efficacy and reducing the toxicity of these agents. Furthermore, determining preclinical and clinical toxicity and safety remain major challenges, due to the different properties between protein-based and small molecule drugs. The high PK variability is clinically important for mAbs, and especially for ADCs, as these agents have a narrow therapeutic index. In addition, the combination of ADCs with other mAbs and immune modulators has a high likelihood of causing drug–drug interactions, as these agents all undergo clearance by the mononuclear phagocyte system (MPS). Thus, the evaluation of biomarkers of the MPS function, Fc receptors, and mediators and drug exposures in MPS cells, are critically important to optimizing the treatment of ADCs alone and in combination with other agents. In this review, we will summarize the factors that have been shown to affect the pharmacokinetics (PKs) and pharmacodynamics (PDs) of mAb and ADC therapies, and the goals of future research to better understand and predict their disposition.

## 2. Formulation Considerations

The use of antibodies as therapeutic agents or targeted carriers is a popular technique in hematology and oncology, as demonstrated by the rapidly growing list of approved antibody-based drugs. However, the majority of these therapies still rely on co-administration with traditional chemotherapy to achieve meaningful clinical responses, and many others have demonstrated lower than anticipated clinical efficacy. Thus, a significant effort has been devoted to enhancing mAb therapies through various modifications, such as ADCs. These immunoconjugates are designed to exploit the specificity of monoclonal antibodies to deliver potent cytotoxic drugs to tumors while limiting off-target exposure. The drugs conjugated to the antibody are highly potent (IC_50_ < 10^−9^ M), and thus, are unable to be safely dosed systemically without a carrier. The four current FDA-approved ADCs are ado-trastuzumab emtansine (Kadcyla^®^, Genentech Inc., South San Francisco, CA, USA), brentuximab vedotin (Adcetris^®^, Seattle Genetics, Inc., Bothell, WA, USA), gemtuzumab ozogamicin (Mylotarg^®^, Pfizer Inc., Philadelphia, PA, USA), and inotuzumab ozogamicin (Besponsa^®^, Pfizer Inc., Philadelphia, PA, USA). Examples of ADCs in clinical trials include mirvetuximab soravtansine (an anti-folate receptor alpha-maytansinoid conjugate) and ABT-414 (an anti-EGFR-auristatin conjugate) [4]. The structure and pharmacology of ADCs are fundamentally different compared to standard small molecule (SM) drugs. These fundamental differences between ADC agents and SM drugs are essential in understanding the differences in PK and PD disposition of these agents.

### 2.1. Monoclonal Antibody Selection

The most common native antibody found in circulation is IgG, and the most commonly used IgG isotype for therapeutic use is IgG1 [5]. The strength of the various effector functions varies depending on the isotype of IgG antibody selected [6,7]. For instance, induction of antibody-dependent cell-mediated cytotoxicity (ADCC) and complement-dependent cytotoxicity (CDC) are stronger with IgG1 and IgG3 isotypes, compared to other isotypes. Whereas, IgG3 is more efficient at inducing tumor cell lysis despite having a relatively shorter half-life compared to IgG1/2/4 [6,7]. IgG4 antibodies also have the unique trait of being able to exchange one-half of themselves with another IgG4, meaning they can form new hybrids in circulation [8]. Finally, IgG2 antibodies have been targeted as potentially favorable for therapeutic use, due to their tendency to form covalent dimers. Dimerization aids in antibody–antibody associations, and can enhance the affinity and internalization of the antibody [6,9]. The proper selection of an antibody is important, especially as part of an ADC, as the antibody can retain native/physiologic functions including immune activation, such as ADCC and CDC, or signal inhibition or modulation [3,5].

### 2.2. Target Antigen

The selection of a target antigen is important, as it allows the antibody to recognize a target on a tumor. An ideal target antigen is highly expressed with limited heterogeneity across a tumor, has low expression on normal tissue, minimal antigen shedding to prevent binding with mAb in circulation, and is well internalized by receptor-mediated endocytosis [3,6]. Baselga et al. reported in patients with lymphoma and prostate cancers that a minimum value of tumor-antigen density is a prerequisite for mAb/ADC efficacy [3]. However, another study has shown that efficacy is independent of antigen density. Perez et al. found evidence that target antigens with low expression can be effective as well [5,6]. This highlights that a desirable cutoff value and minimum threshold for desired antigen density is highly variable and depends on many factors, such as internalization rate and binding affinity [3]. Several recent studies have focused on targeting parts of the tumor microenvironment to help increase the delivery of these agents to the tumor, to provide a more robust anti-tumor effect [10,11,12,13,14].

Generally, experimental evidence would suggest that a correlation exists between available antigen expression and ADC efficacy. However, an in vitro study evaluating an anti-CD22-DM1 ADC against 18 different non-Hodgkin lymphoma (NHL) cell lines showed that sensitivity to the cytotoxic payload (i.e., DM1) was more predictive of response than antigen expression levels [15]. This suggests that patient selection based only on antigen expression may not guarantee anti-tumor efficacy, and additional tumor and non-tumor markers may be necessary to select patients for ADC therapy.

### 2.3. Cytotoxic Payload

Compared to “naked” mAbs, ADCs also contain SM drugs conjugated to the targeting mAb. These cytotoxic payloads are often highly potent, and are targeted to enter the tumor cells by nature of antibody-mediated endocytosis, eventually resulting in the intracellular release of the drug from the mAb carrier [3]. The potency of these agents (in the nM or pM range) typically preclude their use as traditional SM intravenous therapies, due to systemic toxicity [16].

The first generation of ADCs contained payloads that were already traditionally used for chemotherapy, such as doxorubicin and methotrexate, and other microtubule inhibitors and DNA-damaging drugs [3]. Studies proved that first generation ADCs had limited success, with high toxicity, poor chemical properties, and only 1–2% of the drug reaching the target tumor [3,17]. The second generation of ADCs focused on stabilizing linkers and payloads that were 100- to 1000-fold more potent than those in the first generation [18]. Second generation ADCs are exemplified by ado-trastuzumab emtansine (Kadcyla^®^, Genentech Inc., South San Francisco, CA, USA) and brentuximab vedotin (Adcetris^®^, Seattle Genetics, Inc., Bothell, WA, USA), which have a more stable linker between the mAb and drug, and are FDA-approved ADCs. For example, ado-trastuzumab emtansine contains the linker maleimidomethyl cyclohexane-1-carboxylate (MCC), which allows the cytotoxic drug to remain stable in circulation until it is released into the tumor, improving the overall therapeutic index of the ADC (Kadcyla, Genentech Inc., San Francisco, CA, USA). Despite improvements and regulatory success of second generation ADCs complex issues still remain in the selection mAb, linker, and active drug.

### 2.4. Linkers

To attach a cytotoxic payload to a mAb, the use of a chemical linker is required. Linkers are comprised of functional groups, such as disulfides, hydrazones, and thioethers, to connect the mAb and SM drug, and control the release, distribution, and delivery of the cytotoxic agent to the target cell [6,19]. If a drug in released from the mAb carrier prior to delivery to the tumor, it can cause severe off-target toxicities by killing healthy cells. Because these linkers play an essential role in determining the PK and therapeutic index of an ADC, they should ideally be stable enough to allow the ADC to release the drug efficiently, but only at the targeted cells. Hydrophilic polyethylene glycol (PEG) linkers, used as a single chain or arrangement, are particularly attractive as a conjugation linker to improve ADC solubility or reduce ADC aggregation [6,20]. Typically, linkers used in ADC formulations are classified as being either “cleavable” or “non-cleavable”. Cleavable linkers respond to physiological stimuli, such as low pH or proteolytic cleavage, to release the cytotoxic drug from its ADC carrier [21]. In theory, these linkers are catalyzed in a tumor cells, due to the presence, or increased presence, of their catalyst, to allow for selective release of the cytotoxic agent [5,21]. An example of a cleavable linker is seen in brentuximab vedotin, where the monomethyl auristatin E (MMAE) cytotoxic payload is released by protease activity (Adcetris, Settle Genetics, Seattle, WA, USA). On the other hand, non-cleavable linkers rely on lysosomal degradation to release the cytotoxic payload after an ADC has been internalized by the cell [21]. In the lysosomal compartment, the process of intracellular proteolytic degradation reduces the ADC to the level of amino acids. For this type of linker to be effective, the cytotoxic agent must maintain its activity, despite still being attached to part of the linker [22]. ADCs with non-cleavable linkers are considered to have improved therapeutic index because of their greater plasma stability, although new designs of cleavable linkers have significantly improved stability [23].

**Figure 1:**
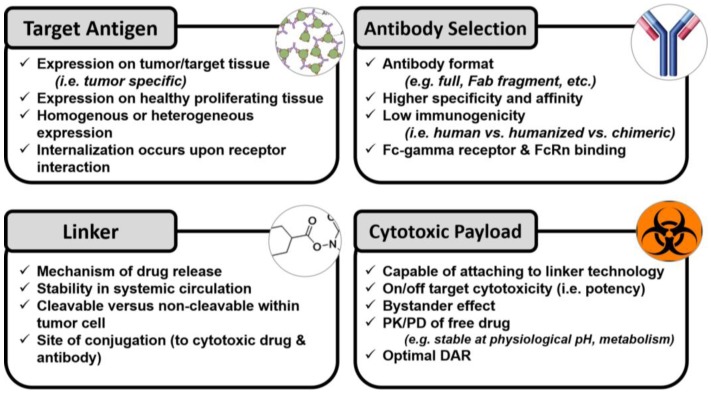
Considerations in the design and development of antibody–drug conjugates that affect the pharmacokinetics and pharmacodynamics of the agents. PK, pharmacokinetic; PD, pharmacodynamic; DAR, drug–antibody ratios.

It is clear that the disposition of a mAb is dependent upon several factors (Figure 1), and not just the therapeutic entity conjugated to it, in the case of an ADC. While mAbs trigger an effector function on their own, ADCs act more like prodrugs, and may or may not exert an anti-cancer effect until the SM drug is released from the mAb carrier. Thus, the pharmacology of mAbs and ADCs are complex. As a result, analytical and PK studies must be performed to assess the disposition of not just conjugated and released forms of ADCs, but also the effects of modifications on the mAbs after administration.

## 3. Pharmacokinetic Considerations

The need to perform detailed PK studies of both conjugated and released forms of drug after administration of an ADC is critical to fully understand the PK and PD disposition of ADCs. An example of this need was demonstrated by gemtuzumab ozogamicin (Mylotarg^®^, Pfizer Inc., Philadelphia, PA, USA), the first ADC to reach the market after gaining FDA approval in 2000. It is made up of an extremely potent anti-tumor antibiotic, calicheamicin, coupled to an anti-CD33 IgG via a pH-sensitive hydrolysable linker. Early trials showed promising remission rates in the high-risk population of older patients with relapsed acute myeloid leukemia (AML) [24]. However, confirmatory trials indicated a higher incidence of early fatality in patients receiving gemtuzumab ozogamicin, and it was voluntarily withdrawn from the market in 2010 [24]. In vitro studies showed that the linker was poorly thermostable, with rapid and extensive release of calicheamicin from the antibody, which resulted in toxicity to CD33-negative MOLT-16 cells [25]. This high systemic exposure of the released active drug was implicated in the significant toxicities associated with administration of gemtuzumab ozogamicin in patients [26]. Continued research using fractionated dosing regimens established improved survival in patients with newly diagnosed AML [27,28]. Positive outcomes of three investigator-initiated trials supported the FDA approval and reintroduction of gemtuzumab ozogamicin with new dosing recommendations in September 2017 [29].

### 3.1. Pharmacokinetic Disposition

**Absorption.** Well known physiological barriers limit oral administration of protein-based drugs and require that most of these agents be administered parenterally to reach systemic circulation [30]. As a result, mAb agents are typically administered either intravenously (iv) or subcutaneously (sc). For oncology indications, iv infusion is the most frequent route of administration for ADCs. By definition, these drugs have 100% bioavailability. By contrast, there are multiple FDA-approved and commonly used mAbs indicated for inflammatory diseases that are administered via sc injection, where bioavailability ranges from ~50–80% following sc administration (Humira, AbbVie Inc., Chicago, IL, USA; Cimzia, UCB Inc., Smyrna, GA, USA; Simponi, Janssen Biotech Inc., Horsham, PA, USA; Xolair, Genentech Inc., San Francisco, CA, USA) [31]. However, sc administration is highly improbable for ADCs, due to the potential reactions to cytotoxic payloads and off-target toxicities mediated by immune cells in the skin, which may cause local deposits of cytotoxic material.

**Distribution.** Due to their size and polarity, the distribution of ADCs is generally restricted to the vascular and interstitial space [32]. Convective transport from blood vessels into tissues is slow and reliant upon pressure gradients [32]. The local structure of both the blood vessel, including fenestration size and membrane thickness, and the surrounding tissue, alters the rate of transport. For example, the tight junctions of blood vessels in the brain effectively limit antibody penetration, resulting in very low distribution of mAbs in brain tissue [32]. By contrast, tumors tend to have leaky vasculature with large pore sizes, allowing increased convective transport of macromolecules into tumors [32]. These are similar barriers that have limited the tumor delivery of other carrier mediated agents, such as nanoparticles and polymer conjugates [33,34]. However, these tumor barriers may be less of an issue for ADCs, as they are smaller (~10 nm) than nanoparticles (~50–100 nm) [35].

Despite this advantage, numerous other factors within tumors may restrict or inhibit the distribution of ADCs. For antibodies with high target affinity, the availability of target antigens near blood vessels may restrict further distribution away from blood vessels due to rapid, tight antigen binding. This phenomenon is known as the “binding site barrier”. Lee and Tannock investigated the distribution of cetuximab and trastuzumab in mouse models of human epidermoid carcinoma (A431) and breast adenocarcinoma (MDA-MB-231) [36]. They found that antibody distribution was dependent on both time and dose administered, and that regions of hypoxia had poor antibody binding. At early time points after dosing, antibody distribution in tumor was heterogeneous and concentrated near blood vessels. Over time, and with increasing dose, the distribution became more homogenous, with the exception of hypoxic tumor regions. Poor binding in hypoxic regions may have been due to low antigen expression and/or decreased local blood flow [36]. Other tumor factors also play a role in the local distribution of ADCs. Characterizing the interactions between tumor factors and ADCs may help to improve the tumor delivery and efficacy of these agents.

ADCs are subject to the same distributive processes and barriers as mAbs, but additionally bear a cytotoxic payload that can further affect their distribution. Both binding affinity and internalization efficiency of the antibody component may be altered by the conjugated entities. These differences may impact distribution hindrance, due to the binding-site barrier described above. In addition, the conjugated SM drug has its own unique distribution profile once cleaved from the antibody. Hydrophobic drugs may be able to cross membranes and distribute beyond the target cell while hydrophilic drugs are generally limited to the antigen-expressing cell that internalized the ADC. Small molecule drugs that have increased permeability and distribution on their own can take advantage of the “bystander effect” and may be beneficial in tumors with heterogeneous target expression. The protein binding characteristics of the released drug (e.g., % non-protein bound drug) also influence the exposure of active drug [37,38]. Breij et al. demonstrated complete tumor regression in patient derived xenograft models of solid tumors following treatment with an anti-tissue factor mAb conjugated to MMAE, despite target antigen expression in just 25–50% of tumor cells [39]. The high response was believed to be due to MMAE to cause a bystander effect [39]. Achieving the ideal combination of antibody and conjugated drug characteristics is therefore important to the efficacy and safety of ADCs, and is likely a unique balance for each target or disease.

**Figure 2:**
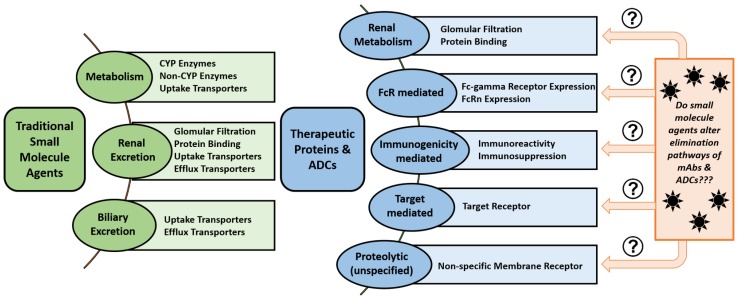
Differences in the metabolism and elimination of small molecules drugs compared to antibodies and antibody–drug conjugates (ADCs).

**Metabolism and Elimination.** The metabolism and elimination of mAbs differs significantly from traditional SM drugs. SMs typically undergo renal elimination or phase I and II metabolism, resulting in metabolites with altered polarity, molecular weight, and activity that may be more easily excreted from the body. Antibody-based therapeutics are cleared via a complex combination of specific and non-specific mechanisms (Figure 2). Degradation of ADCs occurs nonspecifically via proteolysis in a variety of tissues, including the skin, muscle, and liver, due to macrophage uptake [40]. These cells may take up antibodies through non-specific pinocytosis and degrade the engulfed antibodies via lysosomal proteolysis.

ADCs may also be cleared by several specific mechanisms. For antibodies with cellular targets, target-mediated clearance occurs when the antibody binds to the target cell and is internalized and degraded. This clearance pathway is saturable, and may result in non-linear PKs, particularly with low doses and/or high expression of the target. Antibodies also bind to Fc-gamma receptors (FcγR) expressed on cells of the mononuclear phagocyte system (MPS). Interaction with FcγRs also leads to internalization and catabolism of mAbs. This pathway may be particularly important for ADCs with circulating or secreted protein targets, or those that form larger immune complexes, as larger complexes tend to have a rapid and high binding to FcγRs [41].

Rather than attempting to overcome this clearance mechanism, Kasturirangan et al. recently published a report taking advantage of these phagocytic pathways to both neutralize and clear IL-6 with a bispecific antibody [42]. Complexes of a single antibody and single circulating target–antigen complex, such as occurs for IL-6, can evade clearance due to weak FcγR interactions, and extend antigen half-life via the FcRn recycling pathway. To counteract this effect, a bispecific antibody construct was generated from two mAbs targeting different epitopes of IL-6, by joining the single chain variable fragment (scFv) of one antibody with the C terminus of the other antibody. This structure facilitates generation of large branched immune complexes in the presence of IL-6 in vivo. C57BL/6 mice dosed with IL-6 and either individual mAb displayed extended circulation of the mAb-IL-6 complexes. By contrast, mice dosed with IL-6 and the bispecific antibody displayed rapid clearance of the complexes from circulation with FcγR-dependent accumulation in the liver, a primary MPS organ [42]. This novel approach demonstrates the potential for improved drug design based on an understanding of antibody pharmacology and interactions with the MPS.

### 3.2. Mononuclear Phagocyte System

As we strive to better understand the PKs and PDs of antibody-based drugs, it has become apparent that similarities exist between these agents and nanoparticles (NPs; e.g., liposomes, polymeric NPs, micelles, conjugates) (Table 2). Factors affecting the PKs and PDs of NPs are also at work for mAbs, and these mechanisms are ultimately driven by the MPS. The MPS plays a significant role in the distribution, clearance (CL), and activation of mAb and NP drugs. There is also high variability in the function and phenotype of the MPS. In addition, there is a bi-directional interaction between the MPS and NPs, and potentially mAbs, where the physical characteristics of the carrier alters the function of the MPS [43].

NPs and mAbs both demonstrate a high delivery and distribution to the MPS organs, which include liver, spleen, and lung. NPs are well known to accumulate and be cleared through these MPS-associated organs [44,45,46]. Likewise, high relative distribution of trastuzumab to the liver, spleen, and lungs has been observed in mouse models of cancer [47,48]. The CL of NPs and mAbs is attributed to their recognition and uptake by the MPS in these organs. Additionally, high interpatient variability is associated with NP pharmacology. Schell et al. reported a meta-analysis that compared the variability of patients receiving a liposomal therapy with patients receiving the SM counterpart. The results showed a significant increase in PK variability (i.e., plasma area under the curve (AUC)) for liposomes (66%) compared with SM (31%) in patients receiving similar doses [49]. In comparison, the interpatient variability in CL among patients receiving trastuzumab has been reported at 43% (95% CI: 0.213 to 0.238) [50]. Lastly, the CL of NPs is non-linear, likely due to saturation of the MPS [51]. Such saturable CL has also been observed for mAbs as well [52].

Physical characteristics of the drug and of the patient also affect the PKs of NPs and mAbs. NPs demonstrate an accelerated CL when there are a greater number of ligands linked to the carrier, as is the case with actively targeted NPs when compared to non-active NPs. Gabizon et al. have shown that liposomes formulated with folic acid on their surface were cleared faster than the liposome without the folic acid ligand [53]. This is paralleled for ADCs like ado-trastuzumab emtansine, where trastuzumab is conjugated to emtansine, and clears more rapidly (3-fold) in mice and humans than trastuzumab alone [54,55,56]. This is attributed to the ability of the MPS to recognize and take up these more hydrophobic, non-self-agents. Furthermore, a patient’s body habitus has been shown to affect the pharmacology of both NP and mAb drugs. Population PK studies of trastuzumab in patients with HER2+ metastatic breast cancer have shown that body weight is a covariate of CL where increased body weigh correlates with increased CL [50,57]. This is consistent with the altered MPS function for patients with a larger body mass and weight, which have higher MPS function and higher CL of NPs [43]. Finally, tumor burden is also a covariate related to CL of NPs, and increased CL is observed with the number of metastatic sites [58,59]. This same effect is observed with mAb drugs, exemplified in the population PK analysis of trastuzumab, that showed baseline tumor burden was a significant covariate for CL and volume of distribution [50]. MPS function and NP CL are higher in tumor bearing mice compared to non-tumor bearing mice, and in patients with higher tumor burden and certain tumors, especially when tumors are present in liver [58,59,60].

While the MPS may play a role, the importance of this pathway is incompletely understood, and probably differs for different antibodies depending on their antigen specificity and isotype. With the heavy genetic manipulation of mAbs being optimized for higher affinities to targets, improved specificity, and reduced CL rates, an unwanted consequence appears to be an increased incidence of patients demonstrating unexpected or highly variable PK profiles [61,62]. In several studies, common factors known to affect mAbs (target binding, FcRn binding, whole blood stability, anti-drug antibodies (ADA)) were evaluated and shown to not account for the variability in the PKs of mAbs and ADCs [63,64,65]. These results suggest that the variable PK of these agents in certain patients may be due to an increase in low affinity receptor binding, an increased off-target binding and/or inter- and intrapatient variability in MPS function [66].

Two ADCs that preferentially target antigens on tumor cells, that have demonstrated clinical efficacy with manageable safety profiles, are brentuximab vedotin and ado-trastuzumab emtansine [4,67,68]. Both of these agents utilize tubulin-binding agents as their cytotoxic payload, but ado-trastuzumab emtansine uses a non-cleavable linker, while brentuximab vedotin uses a cleavable linker. Both agents incorporate an average DAR of 3.5–4 (though the products are actually heterogeneous with DAR ranging from 0 to 8) [69]. Ado-trastuzumab emtansine has also been shown to have non-specific distribution to highly perfused organs without demonstrating accumulation, similar to the unconjugated “naked” mAb trastuzumab [70]. Brentuximab vedotin demonstrated similar PK results as ado-trastuzumab emtansine above (i.e., ADC and naked mAb tissue distribution profiles), though the ADC seems to present slight accumulation in the liver [71,72]. This data would suggest that certain formulation characteristics of brentuximab vedotin may make it more recognizable to the host’s immune system and MPS compared to the naked mAb, resulting in hepatic accumulation due to MPS-based clearance. However, it is unknown if this hepatic accumulation is the result of ADC linker type (i.e., cleavable and/or not as stable), leading to MPS-related accumulation or that released drug quickly accumulates in the liver.

**Figure 3:**
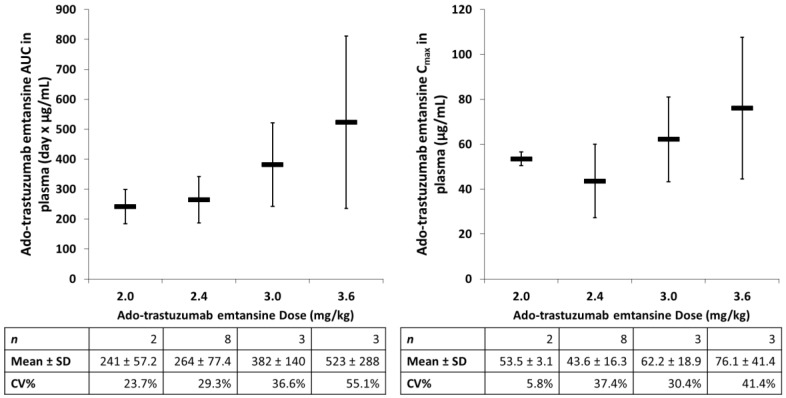
Relationship between ado-trastuzumab emtansine dose and AUC or Cmax in plasma. Mean ± standard deviation (SD) of patients for each treatment group are represented by the black bar. There was high interpatient pharmacokinetic (PK) variability in ado-trastuzumab emtansine, especially when approaching important PK doses (i.e., maximum tolerable dose). The high PK variability of ado-trastuzumab emtansine may be associated with variability in the mononuclear phagocyte system (MPS). CV%, coefficient of variation.

ADCs also demonstrate high interpatient variability and distribution patterns that make understanding the dose–response relationship difficult. The PK of ado-trastuzumab emtansine, total mAb, and DM1 were evaluated in combination with paclitaxel in a phase Ib/IIa trial in patients with HER2-positive metastatic breast cancer administered iv every three weeks [73]. The relationship between ado-trastuzumab emtansine dose and PK parameters in plasma is presented in Figure 3. There was high interpatient PK variability in ado-trastuzumab emtansine AUC and Cmax in plasma, with increasing variability (i.e., CV%) as patients approached the MTD (3.6 mg/kg). Another ADC, anetumab ravtansine (BAY 94-9343), is a novel fully-human anti-mesothelin immunoglobulin G1 antibody conjugated to the maytansinoid tubulin inhibitor DM4. Anetumab ravtansine AUC in plasma demonstrates similar high interpatient variability, which was found to not be associated with anti-drug antibody (ADA) titers [74]. In addition, there was an overlap of anetumab ravtansine plasma exposures at doses of 5.5, 6.5 (MTD), and 7.5 mg/kg. The high PK variability of these ADCs may be associated with variability in the MPS, especially considering a lack of a relationship between PK variability and ADA titers.

## 4. Physical Characteristics of ADCs

### 4.1. Size

Therapeutic proteins come in a range of sizes, and differences can affect the PK disposition in various ways. Examples such as full IgG mAbs, Fab fragments (Fabs), serum albumin, and streptavidin are just a few that have been examined. These proteins were injected into SCID mice bearing HeLa tumor xenografts, and blood concentrations were measured over the course of 7 days [75]. Full IgGs had the slowest clearances compared to streptavidin and body surface area (BSA), and Fabs had the highest clearance, which was directly related to its molecular weight [75]. While Fab fragments are one-third the size of full IgG antibodies (~50–66 kDa vs. ~150 kDa, respectively), the clearance is 10-fold greater (1.1 mL/h vs. 0.1 mL/h) though the Vd is similar between all sizes (~1.5 mL) [75]. The clearance value for Fabs is greater because these molecules are smaller than IgGs, and as such, they are also cleared in more traditional pathways (i.e., hepatic excretion and renal elimination) than via cellular interaction and endocytosis, such as interaction with the MPS or proteolytic degradation. In terms of pharmacological benefit, larger proteins (full IgG mAbs or ADCs) could increase the amount of time an agent remains in the body, leading to prolonged dosing intervals and increased time of exposures, which leads to prolonged therapeutic effect.

Conjugating SM drugs to antibodies has been shown to be safe and effective, with regards to patient care, though few examples exist of smaller proteins (such as Fabs) as carriers. The Chinese Food & Drug Administration (CFDA) previously approved Metuximab-I^131^, a murine IgG1 anti-CD147 F(ab)2 conjugated to iodine-131, for the treatment of liver cancers. Metuximab-I^131^ had a longer half-life when compared to the naked Fab fragment (1.96 h vs. 0.6 h, respectively) [76]. Similarly, naptumomab estafenatox (an anti-5T3 Fab conjugated to a staphylococcal enterotoxin) has a terminal half-life of 1.38 h, demonstrating similar PK to that of Metuximab-I^131^ [77]. Based on these data, conjugated Fabs have a lower clearance and longer terminal half-life values compared to “naked” Fabs. This may be due to a reduction in the ability or rate of traditional clearance pathways, or the impact of additional clearance pathways due to the increased size of the agent based on the size of the conjugated drug. In addition, PK of Fab-ADCs may not be affected by concomitant chemotherapy compared to full mAbs, as naptumomab estafenatox administered in combination with docetaxel resulted in a non-significant change in half-life [77].

### 4.2. Drug–Antibody Ratio (DAR)

The drug–antibody ratio (DAR), or number of drug molecules conjugated to a single ADC, is critical in determining efficacy. Optimal DAR to achieve the greatest clinical efficacy has yet to be determined, and may be highly dependent on other ADC variables. In addition, DAR varies within a single product, and controlling this heterogeneity is complicated, due to the number of lysine residues exposed on the antibody surface, such as for ado-trastuzumab emtansine, and the cysteine residues used in creating interchain disulfide bonds, such as for brentuximab vedotin [3]. With too few molecules attached, the ADC may not provide an adequate cytotoxic response or anti-tumor efficacy [3]. On the other hand, too many drug molecules conjugated to an ADC can become unstable, be rapidly recognized by the immune system, produce altered PK and PD properties, and increased toxicity [78].

It has been shown previously that a higher DAR leads to greater in vitro potency, though the increased DAR also affects the pharmacological properties of the agent [78,79]. For instance, the conjugation of MMAE in the creation of ADCs, or doxorubicin in high DAR ADCs, resulted in increased aggregation, both believed to be due to their added hydrophobicity [78,80,81]. To offset the hydrophobic detriments of cytotoxic payloads, hydrophilic-based polymer linkers are being evaluated. Preliminary studies of these hydrophilic linkers show that higher DARs (up to a DAR of 20) increase potency, but also maintain the desired biological/pharmacological properties in vivo [79,82].

**Figure 4:**
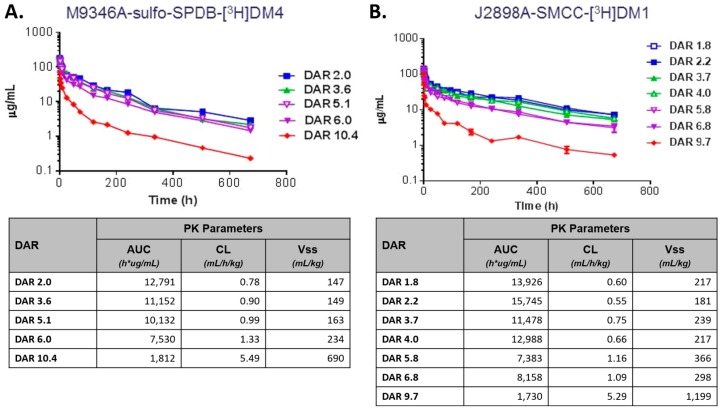
Pharmacokinetic studies evaluating antibody–drug conjugate (ADC) concentrations using radiolabeled ADCs. Clearance of M9346A–sulfo-SPDB–[3H]DM4 (**A**) and J2898A–SMCC–[3H]DM1 (**B**) conjugates from plasma of CD-1 mice, that were injected iv as a single 10 mg/kg dose, were measured by counting the radioactivity in plasma arising from the tritium label on the maytansinoid. These findings suggest that maytansinoid conjugates, regardless of linker type, with drug–antibody ratios (DAR) ranging from 2 to 6, have a better therapeutic index than conjugates with very higher DAR (>9). Adapted with permission from [79]. Copyright 2017, American Chemical Society.

There have been extensive studies on the effects of DAR on the properties of ADCs. One group prepared a series of maytansinoid-coupled ADCs with a DAR range from 2 to 14, using both cleavable and non-cleavable linkers, to evaluate the preclinical and clinical effects of DAR, including in vivo stability, efficacy, and tolerability [79,83]. Their results showed that agents with a high DAR (average 10, range 7–14) using both linker formats had faster distribution and clearance rates and decreased efficacy in vivo (Figure 4) [79,83]. On the other hand, ADCs with a DAR less than 6 displayed similar clearances, superior in vivo efficacy, and were well tolerated [79,83]. Hambelet et al. demonstrated similar conclusions in a study evaluating MMAE conjugated to a CD30 antibody using a MC–vc–PAB linker [78]. When DARs were manipulated to increase the cytotoxic payload with either 2, 4, or 8 drugs per antibody, the clearance increased [78]. These results may be related to the difference in the rate of uptake of these agents by the MPS and other non-target cells.

Additionally, the site of conjugation can play a role in augmenting the pharmacology of an ADC. Strop et al. created an ADC with an average DAR ~1.7, but directed conjugation using a novel enzymatic conjugation to precise points on the antibody backbone to create homogenous solutions [84]. Their study reported how the site of conjugation affected linker stability in a species-dependent manner, where conjugation on the light chain appears to be cleaved more quickly compared to heavy chain conjugation in mice, but stable in rats [84]. Additionally, heavy chain conjugation demonstrated dramatically different PK and faster rate of drug loss, compared to either light chain conjugated ADCs or naked mAbs in rats [84]. The uses of such technologies can allow for increased studies of the role of the linker, linker position, and cytotoxic payload to optimize the pharmacology and improve the therapeutic index of these agents.

### 4.3. Surface Modifications

Modifying formulation and structures of drug carriers is a common practice as a method to alter the way the body handles an agent. The most common way to modify these agents is by glycosylation and PEGylation. These modifications can be made on the antibody or the linker, and may reduce the rate of clearance.

**Glycosylation.** The post-translational modification of proteins through the addition of carbohydrates (i.e., glycans) to amino acid side chains is a process known as glycosylation. This natural modification has been associated with the production of therapeutic proteins in eukaryotic cell lines and is dependent on several factors, including the cell line and culturing conditions [85]. However, both the amount and location of glycosylation dramatically affects the disposition of these proteins, such as regulating receptor binding and Fc effector functions [86]. In addition, *N*-linked glycosylation has been used for promoting the systemic residence time, especially for smaller proteins like diabodies [87]. While conflicting studies have shown that glycosylation of the Fc region may affect clearance, it is generally acknowledged that high mannose-5 glycan forms may be cleared more rapidly compared to other glycoforms [88,89]. Ado-trastuzumab emtansine was glycoengineered to include terminal sialic acids [90]. This allows for payload addition without remodeling the entire antibody to avoid potential interference with drug loading. However, the analysis of heterogeneous populations (due to different levels of glycosylation and glycoforms) makes thorough analysis of different drug formulations difficult to compare.

**PEGylation.** PEGylation, the addition of non-immunogenic PEG polymer, is another suitable option for modifying antibodies to overcome disadvantages of certain biologics. In general, PEGylation provides improved agent solubility, decreased immunogenicity, and prolonged residence time in the body [91,92,93]. This occurs as PEG protects against enzymatic degradation, slows filtration by the kidneys, and slow recognition by the MPS [94]. However, PEGylation can also influence binding affinity/potency to its target, due to steric limitations, thus, individual conjugation sites and control of conjugation should be evaluated. The main use of PEGylation on ADCs is as a linker to improve the solubility and limit aggregation. In a study by Burke et al, drug-like PEG side chains were examined to determine PK parameters [95]. The group used a dose of 3 mg/kg MMAE in Sprague-Dawley rats, and monitored the amount of antibody over time. The length of the PEG-chain was varied from 2 to 24 PEG-block polymers, and each ADC was conjugated with 8 MMAE drug molecules [95]. After iv administration in the rats, the increased length of the PEG-chain resulted in lower clearance [95]. This relationship held true for PEG_2_ and PEG_4_, but from PEG_8_–PEG_24_ there were only minor differences between the PK parameters [95]. Using PEG on Fab fragments also increased the circulation time [93].

### 4.4. Charge and pH Engineering

The charge of a protein is a critical variable in determining its electrostatic/non-specific interactions with other materials found within circulation and tissues. A fundamental property of proteins is their isoelectric point (pI), or the pH at which the antibody carries no net electrical charge, and traditionally falls in a range between 8 and 9 [96]. However, due to current manufacturing and isolation techniques, ADCs will be heterogeneous in relation to the surface charge and pI properties within the entire protein pool. Shifts in the isoelectric point of even one pI unit can produce measurable changes in tissue distribution and kinetics [97,98]. Cationization (where the pI is raised/more basic) of antibodies have been associated with increased blood clearance and higher tissue accumulation, tending to adhere to more anionic sites on the cell surface [96]. One study assessed the effect of increasing the pI of the antibody by 2 units (from pI 7 to 9) and found a 28-fold increase in plasma clearance [99]. However, minor changes to the pI (typically < 1.0 unit) did not appear to affect mAb function or PK, suggesting that minor changes in formulation pI may not warrant additional PK considerations [96,100]. Cationization of the antibody drug has also been used to encourage extravasation, antigen binding, and receptor-mediated endocytosis of antibodies into the target cells due to electrostatic interactions (positively charged antibody and the negatively charged cell membrane), resulting in non-specific membrane flow [96,101,102]. Therefore, it is important to isolate and characterize charge variants, and evaluate the PK disposition of specific isolates, as variability in PK could be due to a heterogenous pool of agents.

## 5. Host-Associated Factors and Disease Status

### 5.1. Sex and Body Habitus

Historically, it is controversial if sex and/or body weight play a role in the disposition of mAb agents, though examples do exist. A population analysis of 671 patients enrolled in the five phase I to III studies of ado-trastuzumab emtansine found that serum albumin, serum AST, tumor burden, and body weight all demonstrated significant effects on ado-trastuzumab emtansine PK [103]. Of these factors, body weight demonstrated the greatest effect, where patients with higher baseline weight had larger effects on both CL and Vc [103,104]. These data also proved to be the most sensitive in affecting the steady-state AUC and Cmax concentrations of drug [103]. Similar effects were detected for brentxumiab vedotin in a population PK analysis of 314 patients evaluated in five trials. In this evaluation, body weight and BSA affected clearance and volume of distribution [105]. In both cases, patients with larger body habitus had a higher CL and lower exposure of the agents. Both of these studies support the decision to utilize weight-based dosing strategies for these ADCs, though variability still exists despite weight-based dose normalization. The higher CL in patients with larger body habitus and the inability of weight-based dosing to fully account for the differences in PKs are consistent with higher MPS function in overweight and obese patients [43].

### 5.2. Chemical Modulators of Immunity in Blood

While traditional population PK studies have routinely used patient covariates to evaluate PK differences across patient populations, various blood chemistry factors know to regulate immunity and tissue effectors have also been evaluated. As the MPS appears to play a significant role in the disposition of mAb agents, sex hormones and chemokines that regulate the MPS may also affect the PK disposition of these agents similarly [106,107,108]. Several studies have demonstrated that sex hormones have in vitro regulatory effects on lymphocyte and macrophage functions, including macrophage FcɣR expression, which can lead to increased clearance of IgG-coated materials [109,110,111,112,113,114,115]. Certain hormones, such as calcitriol, the most potent form of vitamin D3, also appear to be important mediators of FcɣRs and function, and have previously been shown to regulate key immune cytokines in mononuclear phagocytes (IL-1, IL-6, TNFa) and T lymphocytes (IL-2, INFg) [116,117,118,119,120,121]. These effects may be more prominent in tissues, where activated macrophages can be affected by locally produced calcitriol and cytokines, reaching pharmaceutically-relevant concentrations and influencing cellular immune responses.

### 5.3. Renal or Hepatic Impairment

According to the FDA, the assessment of mAb agents in special populations, such as renal or hepatic impairment, is not necessary [122,123]. This is because renal and hepatic impairment is not likely to alter the PK disposition because the excretion by the kidneys and liver are not significantly involved in the clearance and disposition of mAbs agents. In addition, case reports in patients undergoing hemodialysis have reported that renal impairment did not affect the PKs of bevacizumab, cetuximab, rituximab, or trastuzumab [124,125,126]. While most agents may not be affected, the kidneys may play a role in the elimination of agents capable of passing glomerular filtration (~<60 kDa) [127]. These agents that pass the cutoff typically experience a gradual decrease in their clearance, and thus increased exposure, with increasing renal impairment [127]. However, Czock et al. reported 3-fold reduction in CL of low molecular weight peptides and proteins (<50 kDa) in patients with severe renal failure or end stage renal disease (ESRD) [128]. Future studies are needed to determine the effects for smaller antibody fragments (e.g., Fab, scFv, adAb) and with conjugation to form ADCs, as cytotoxic payloads provide additional mechanisms of alteration.

### 5.4. Neonatal Fc Receptor (FcRn)

The role of FcRn in supporting the prolongation of serum IgG half-life in circulation has been well characterized [129]. Its existence was discovered in the 1960s as a transporter of maternal antibodies to a fetus, thus its original name of the neonatal Fc receptor (FcRn) [130]. This receptor is widely expressed within cells of various organs throughout the body [131]. FcRn does not bind to its ligand (i.e., “Fc region” or CH2–CH3 interface) at physiological pH (pH = 7.0–7.5), but only in the acidic environment of an endocytic vacuole (pH < 6.5) when exposed histidine residues on FcRn become protonated and increase their affinity to the Fc region of IgGs [132]. It is generally believed that serum IgGs are first internalized via various pathways (nonspecific endocytosis or fluid-phase pinocytosis) into an intracellular endosome, before they can bind to FcRn to be recycled and expelled out of the endosome and cell [131].

As mentioned previously, FcRn contributes to the overall PK profile of mAb-based therapeutics in several potential ways. FcRn is widely expressed throughout the body, including within parenchymal and hematopoietic cells in fat, kidneys, liver, muscle, skin, spleen, and placenta, but may have differing functions based on the tissue. Chen et al. examined the effect of FcRn on biodistribution of an Fc-containing IgG1 in wild type (WT) and FcRn-knockout (KO) mice [133]. The fat, muscle, and skin of KO mice had a decreased tissue/blood exposure ratio versus WT mice, while the kidney, liver, and spleen had an increased ratio. The differing effects on tissue/blood exposure ratios of FcRn knockout may be explained by differing functions of FcRn in each tissue. For example, in liver and spleen, FcRn may primarily serve to transport antibodies out of tissue back into systemic circulation, while in muscle and skin, FcRn may be responsible for delivering antibodies from circulation to the tissues [133].

Several discussions have been published on how optimization of the interaction with FcRn may alter intracellular transport, thus leading to longer half-lives (and reduced clearance) of mAb-based drugs. Modulation of the FcRn–IgG interaction, to improve PK parameters, has been a strategy used by several investigators to either extend (improving efficacy and reducing dosing frequency) or shorten (for diagnostic evaluation or control for known toxicities) the half-life of an agent. The correlation between FcRn binding affinity and systemic half-life has been investigated extensively for a number of mAbs [66,134,135,136,137,138]. Several reviews have also discussed how mutations in the Fc region affect the FcRn–IgG interaction, and are directly related to observed differences in half-life [136,139,140,141]. Five mutations of note have been previously reviewed, and have been demonstrated to extend serum IgG half-life [142,143]. However, other studies contradict these reports, raising questions on the interaction between FcRn and in vivo clearance [138,144,145]. Cross-species differences in the FcRn–IgG interaction have been evaluated to determine the relevance in the use of preclinical models, especially mice. Because of a stronger affinity of human IgG antibodies for murine FcRn (~15-fold greater than human FcRn), human mAbs exhibit slower clearance and longer half-lives in mice, making them poor predictors for allometric correlation to human clearance rates [144,146,147,148]. This leads to a reliance on genetically engineered mouse models (GEMMs), such as transgenic FcRn murine models, over WT strains in preclinical PK studies, though in vitro or ex vivo analysis would be more efficient for high-throughput comparisons [149,150]. Improved high throughput methods would be of particular benefit, as it is suggested that ~15% of phase I studies fail due to unfavorable PK and PD properties [151].

### 5.5. Fc-Gamma Receptors (FcɣR)

The expression profile of FcɣRs in various tissues is an additional consideration for thorough evaluation of an antibody-based agent. The MPS serves as a natural mechanism of clearance for antibodies and immune complexes via their FcɣR [152,153,154]. Myeloid cells express various forms of FcɣR that will interact with extracellular monomeric or aggregated IgGs, complexes, opsonized substances, and therapeutic mAbs [152]. However, FcɣRs have differing affinities for the various arrangements of IgG [155]. For instance, FcɣRI (CD64) can bind to monomeric IgG via high affinity receptors (~10^7^) while other receptors, such as FcɣRII (CD32) and FcɣRIII (CD16), mainly bind to aggregated IgGs at a lower affinity (~10^6^ to ~10^4^) [152,155,156,157]. Due to the differences in types and affinity of FcɣRs, variations in receptor expression can lead to significant differences in the MPS’s ability to clear immune complexes from the blood. This also translates to variation in the MPS’s ability to take up mAbs and ADCs, which would affect ADC PK and PD.

FcɣRs vary within preclinical models, and these differences need to be considered when evaluating an agent’s PK and making comparisons to human physiology. For instance, both humans and mice carry FcɣRI on myeloid cells and FcɣRIII on NK cells [158,159]. However, mice carry an additional receptor type not found in humans, FcɣRIV, an activating receptor found on neutrophils, monocytes/macrophages, and dendritic cells that binds to IgG2a/b, but not IgG1 or IgG3 [158,159,160]. In NHPs, only a single FcɣRIII gene exists, with homology similar to human FcgRIIIa [158]. However, even within NHP models, expression variability exists. Similar to humans, sooty mangabeys express FcɣRIII on monocytes, neutrophils, and some lymphocytes; however, FcɣRIII is not detected on neutrophils in macaques and baboons [161]. Additionally, NHP FcɣRIII interacts with IgG1 and IgG2, but human FcɣRIII interacts with IgG1 and IgG3 [161]. This makes selection of certain preclinical models obsolete when attempting to determine first in human doses and toxicity profiles.

Abuqayyas et al. reported that the PK of mAbs are not substantially affected by FcɣR expression [162]. In this study, the PK of an IgG1 agent at 0.04–0.4 mg/kg was evaluated in control mice, FcγRI/RIII knockout mice, and FcγRIIb knockout mice. The plasma clearance of the IgG1 was similar for all doses and in all groups. However, the doses of the IgG1 agent were 100-fold and 250-fold lower than “therapeutic doses” of pertuzumab (30 mg/kg) and trastuzumab (100 mg/kg), respectively, required in murine models [163,164]. Thus, there is a high likelihood that the lack of an effect of FcɣR expression on the plasma PK of the IgG1 agent is due to the use of “micro-doses” in this study. Interesting, there was a higher exposure of IgG1 in the liver (an MPS organ) in the knockout mice compared to wild type mice [162]. Thus, even at “micro-doses” of mAbs, FcɣR expression does affect their tissue PK. Studies have also reported that MPS function and FcɣR expression and profiles in mice are different than in humans [108,152,155,165,166,167]. Thus, the preclinical studies by Abuqayyas et al. do not definitively show that MPS FcɣRs do not affect mAb/ADC PK, and further studies at clinically relevant doses are necessary.

## 6. Pharmacologic-Associated Factors

### Drug–Drug Interactions (DDIs)

Assuming classical elimination pathways, traditional SM drugs present a low potential for DDIs with mAbs/ADCs, as these classes of agents follow different pathways of elimination, unless the SM drugs alter the function or are cytotoxic cells involved in the clearance of mAbs/ADCs (Figure 2). It is also possible for the SM drug payloads to affect the immunogenicity of therapeutic proteins [168].

Pertuzumab (Perjeta^®^) is a novel humanized recombinant mAb directed against HER2 with a distinct mechanism of action from trastuzumab. To evaluate potential DDIs associated with co-administration of other agents with pertuzumab, phase I studies evaluating PK of pertuzumab given at the same dose, alone and in combination with trastuzumab and docetaxel, in patients with metastatic breast cancer, were compared [169,170]. The mean serum Cmin for pertuzumab after administration, alone or in combination with trastuzumab and docetaxel, were 35.0 µg/mL and 63.6 µg/mL, respectively. The ~2-fold reduction in clearance and increase in exposure of pertuzumab suggests that combining mAb agents results in a DDI that alters the PKs and PDs of mAbs or ADCs.

Rilotumumab is a fully human monoclonal antibody against hepatocyte growth factor (HGF) and the only known ligand for the MET receptor [171]. The PK of rilotumumab has been evaluated in several trials now, in combination with other traditional anti-cancer therapies and other targeted agents [172,173,174,175]. A recent review of the preclinical and clinical data, to date, of rilotumumab states that DDIs do not exist when co-administered with other therapeutic proteins [171]. However, reference PK studies evaluating monotherapy with panitumumab showed an AUC of 34,100 ± 9260 h∙ug/mL and Cmax of 227 ± 36.7 µg/mL, while dual therapy of panitumumab and rilotumumab at the same dose results in an AUC of 61,400 ± 1620 h∙µg/mL and Cmax of 346 ± 88.6 µg/mL [171]. This is an approximately 80% increase in AUC exposure and 52% increase in Cmax [171]. This study also demonstrated altered PK of rilotumumab when combined with other mAbs. The mean plasma AUC of rilotumumab monotherapy on cycle 5 and rilotumumab at 10 mg/kg plus 10 mg/kg bevacizumab were 55,900 µg/mL∙h and 82,700 µg/mL∙h, respectively—an increase in serum AUC of 47% after the addition of bevacizumab [171,172]. Further studies are needed to determine if these sizeable increases in AUC are clinically relevant.

However, in comparison to SM drugs, there are far fewer evaluations of how therapeutic antibodies may affect the other ADC PK and/or PD. Data with the use of an ADC in combination with other antibodies have only recently begun to be evaluated for efficacy and synergistic effects in preclinical models. Thus, while data is still lacking with ADCs, our growing knowledge of DDIs after administration of multiple antibodies would suggest this is an important factor to evaluate in preclinical models or formulation selection.

## 7. The Next Generation of ADCs

Several hurdles remain for antibodies and ADC to overcome, such as low delivery efficiency, heterogeneity of target antigen expression, and target expression on normal tissues. While the success of maytansinoid and auristatin ADCs is notable, further research is being performed with even more potent cytotoxic compounds (EC_50_ in the pM range) and novel mechanisms of action, as a means to increase the therapeutic window. Of note, pyrrolobenzodiazepine (PBD) dimers and duocarmycin analogs have demonstrated cytotoxicity at 10-fold lower concentrations than those of maytansinoid and auristatin ADCs [176,177]. An example in development is SGN-CD70A, a PBD-conjugated anti-CD70 ADC for the treatment of renal cell carcinoma and NHL, which showed anti-tumor activity in preclinical mouse models when dosed at 0.1–0.3 mg/kg of ADC [178]. On the other hand, a HER2 conjugated duocarmycin analog (vc–seco–DUBA) has shown tumor growth inhibition in patient derived xenografts, despite low HER2 expression, after a single dose of 1 mg/kg [179]. There has been skepticism about the utility of these drugs due to their short half-life (~1 h) after release, but this characteristic may ultimately be of benefit as potential systemic toxicities would be expected to be minimal [180].

There is a considerable emphasis being placed on the optimization and understanding of DAR effects. Of note, substantial effort is currently focused on determining how to direct the site(s) of conjugation and create more homogenous products with a narrow DAR range. THIOMAB was the first approved that used this particular strategy, utilizing strategic cysteine residues [4]. Others have begun to add sequence tags, such as SMARTag and TG-ADC, to direct enzymatic drug conjugation [181,182,183]. Beyond even the number of conjugation sites on a particular carrier, optimal conjugation site selection must also be considered, as site accessibility and linker stability were inversely correlated with intermediate accessible sites in demonstrating increase efficacy and safety [184]. While advancements are creating even higher homogenous DARs, it is still unknown if this leads to an improved therapeutic window while also increasing efficacy/safety.

Instead of altering the cytotoxic payload, others have altered the antibody carrier to affect function, such as using smaller antibody fragments (e.g., scFv, minibodies, sdAb, diabodies). While this may result in increased drug delivery into the tumor microenvironment and provide greater clinical effects, these ADCs will also result in both faster clearance and larger volumes of distribution, changing how we administer these biologic agents from other antibodies and ADCs. Additional studies will provide insight if more frequent dosing of these smaller antibody agent can provide similar or improved clinical efficacy while being more tolerable due to their more rapid clearance.

## 8. Conclusions

The addition of ADCs within the field of medicine has led to great advances in our fight with treating hematologic and solid tumor malignancies, but significant challenges still remain to be solved. However, there are still several more innovative and novel formulations under development and in clinical trials, as immunotherapies begin to take focus. In addition, the use of predictive biomarkers or screening tools other than time-intensive IHC methods will be essential, to ensure effective treatments are targeted towards patients who will gain the most benefit.

While progress has been made in gaining knowledge on the PKs and PDs of mAbs and ADCs, increased understanding of the mechanistic aspects of their disposition and how this relates to efficacy and safety are needed. There are many properties that make ADCs unique compared to an active SM drug. However, antibody-based therapies may also have new toxicities associated with enhanced distribution to specific organs and toxicities associated with the components of the carrier. It has been shown that physical properties, the MPS, host-associated factors, and pharmacologic-associated factors all contribute in varying degrees to altering the PKs and PDs, in preclinical models and in patients. Thus, the pharmacology of ADCs is highly complex. Thus, several challenges still exist to optimize ADC therapies before the field can make further improvements, including DAR coupling strategies, the limited tumor penetration of these agents, and the development of resistance.

There is still much to learn about the clinical applications of biologic-based therapies, but the success of early agents, such as Kadcyla and Adcetris, have emboldened further research into improved treatments and optimizing outcomes based on personal phenotypes. Areas of research that can aid in our understanding of how these agents are handled and how we may predict their actions in patients, include pharmacogenomics, cellular function (probing the MPS), more sensitive and accurate analytical PK methods for determining drug and carrier components, and coupling of phenotypic probes with relevant PK and PD models.

## Figures and Tables

**Table 1:** Antibody–drug conjugates approved and under investigation (Phase II or higher).

**Generic Name**	**Brand Name**	**Manufacturer**	**Phase/Studies Open**	**Target Antigen**	**Linker**	**Payload**	**Indications**
Brentuximab vedotin	Adcetris	Seattle Genetics	Approved	CD30	Cleavable (protease)	MMAE	Hematological
Gemtuzumab ozogamicin	Mylotarg	Pfizer	Approved	CD33	Cleavable (acid labile)	Calicheamicin	Hematological
Inotuzumab ozogamicin	Besponsa	Pfizer	Approved	CD22	Cleavable (acid labile)	Calicheamicin	Hematological
Trastuzumab emtansine	Kadcyla	Genentech	Approved	HER2	Non-cleavable	DM1	Solid
**Generic Name**	**Investigational Name**	**Manufacturer**	**Phase/Studies Open**	**Target Antigen**	**Linker**	**Payload**	**Indications**
Mirvetuximab Soravtansine	IMGN-853	ImmunoGen	I, II, III	FOLRI 1	Cleavable (disulfide)	DM4	Solid
Polatuzumab vedotin	DCDS-4501A	Genentech	I, II, III	CD79b	Cleavable (protease)	MMAE	Hematological
Rovalpituzumab tesirine	SC0001-SCX	Stemcentrx	I, I/II, II, III	DLL3	Cleavable (protease)	SCX	Solid
Sacituzumab govitecan	IMMU-132	Immunomedics	I/II, II, III	TROP2 EGP1	Cleavable (acid labile)	SN-38	Solid
-	AGS-16C3F	Agensys	II	AGS-16/ENPP3	Non-cleavable	MMAF	Solid
Denintuzumab mafodotin	SGN-CD19a	Seattle Genetics	II	CD19	Non-cleavable	MMAF	Hematological
PSMA ADC	-	Progenics	II	PSMA	Cleavable (protease)	MMAE	Solid
Anetumab Ravtansine	BAY 94-9343	Bayer Healthcare	I, I/II, II	Mesothelin	Cleavable (disulfide)	DM4	Solid
Depatuxizumab Mafodotin	ABT-414	Abbvie	I, II	EGFR	Non-cleavable	MMAF	Solid
Enfortumab Vedotin	ASG-22CE	Astellas Pharma	I, II	Nectin 4	Cleavable (protease)	MMAE	Solid
Glembatumumab vedotin	CDX-011	Celldex	I/II, II	gpNMB	Cleavable (protease)	MMAE	Solid
Labetuzumab govitecan	IMMU-130	Immunomedics	I, II	CEACAM5	Cleavable (acid labile)	SN-38	Solid
Tisotumab Vedotin	HuMax-TF	Genmab Seattle Genetics	I/II, II	Tissue Factor	Cleavable (disulfide)	MMAE	Solid
-	CDX-014	Celldex	I/II	TIM-1	Cleavable (disulfide)	MMAE	Solid
-	CX-2009	Cytomx	I/II	CD166	Cleavable (protease)	DM4	Solid
-	DT2219ARL OXS-1550	GT Biopharma	I/II	CD19 & CD22	Cleavable (protease)	Modified diphtheria toxin	Hematological
-	HuMax-AXL	Genmab	I/II	AXL	Cleavable (protease)	MMAE	Solid
Indatuximab ravtansine	BT-062	Biotest	I/II	CD138	Cleavable (disulfide)	DM4	Hematological
Pinatuzumab vedotin	DCDT-2980S	Genentech	I/II	CD22	Cleavable (protease)	MMAE	Hematological

Abbreviations: MMAE, monomethyl auristatin E; DM1, mertansine; DM4, ravtansine; SCX, tesirine; MMAF, monomethyl auristatin F.

**Table 2:** Summary of pharmacokinetic (PK) and pharmacodynamic (PD) similarities for nanoparticles (NPs) and antibody–drug conjugates (ADCs) associated with the mononuclear phagocyte system (MPS) clearance.

	Characteristic	Cause	Example
**1**	High delivery and distribution to MPS organs	MPS cells are involved in the distribution and capture of these agents in liver and spleen.	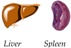
**2**	Faster clearance is associated with agents that have a greater number of ligands linked to the carrier.	MPS is able to recognize and take up these “non-self” agents to a greater extent.	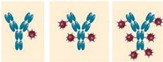
**3**	High interpatient PK and PD variability	MPS function is highly variable in patients	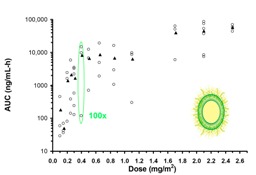
**4**	Non-linear/saturable clearance at high doses.	MPS uptake of particles has a maximum capacity that can be saturated	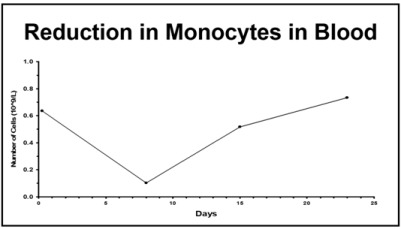
**5**	Body weight, body composition, body habitus are covariates related to clearance.	MPS function is altered in patients with large body mass and weight	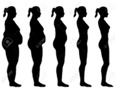
**6**	Tumor burden is a covariate related to clearance	MPS function is increased in patients & animals with large tumor burden, especially when tumors are present in the liver.	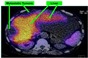

## Abbreviations

ADA	anti-drug antibodies
ADC	Antibody–drug conjugate
ADCC	antibody-dependent cell-mediated cytotoxicity
AML	acute myeloid leukemia
BSA	body surface area
CDC	complement-dependent cytotoxicity
CEA	carcinoembryonic antigen
CFDA	Chinese Food & Drug Administration
CL	clearance
DAR	drug–antibody ratio
DDI	drug–drug interaction
DLT	dose limiting toxicity
ESRD	end stage renal disease
Fabs	Fab fragments
FcγR	Fc-gamma receptors
FcRn	neonatal Fc receptor
GEMMs	genetically engineered mouse models
HGF	hepatocyte growth factor
iv	intravenously
KO	knockout
mAb	monoclonal antibody
MCC	maleimidomethyl cyclohexane-1-carboxylate
MMAE	monomethyl auristatin E
MPS	mononuclear phagocyte system
NHP	non-human primate
NP	nanoparticle
OS	overall survival
PBD	pyrrolobenzodiazepine
PD	pharmacodynamic
PEG	polyethylene glycol
PFS	progression-free survival
pI	isoelectric point
PK	pharmacokinetic
sc	subcutaneously
scFv	single chain variable fragment
SM	small molecule
WT	wild type
